# Aggression and violence against health care workers in Germany - a cross sectional retrospective survey

**DOI:** 10.1186/1472-6963-10-51

**Published:** 2010-02-25

**Authors:** Simone Franz, Annett Zeh, Anja Schablon, Saskia Kuhnert, Albert Nienhaus

**Affiliations:** 1 Institute of Nursing Science at the University of Bielefeld, Bielefeld, Germany; 2 Institution for Statutory Accident Insurance in the Health and Welfare Services, Department of Occupational Health Research, Hamburg, Germany

## Abstract

**Background:**

Although international scientific research on health issues has been dealing with the problem of aggression and violence towards those employed in health care, research activities in Germany are still at an early stage. In view of this, the aim of this study was to examine the frequency and consequences of aggressive behaviour towards nurses and health care workers in different health sectors in Germany and to assess the need for preventive measures.

**Methods:**

We conducted a cross-sectional retrospective survey. Nurses and health care workers from two nursing homes, a psychiatric clinic and a workshop for people with disabilities were interviewed using a standardised questionnaire. The sample covered 123 individuals (response rate 38.8%). The survey assessed the frequency, the type and the consequences of aggressive behaviour, and social support in connection with coping with aggression in the workplace. Odds ratios (OR) and 95% confidence intervals (CI) for putative risk factors which may influence the stress induced by aggression at the workplace were calculated using conditional logistic regression.

**Results:**

During the previous twelve months 70.7% of the respondents experienced physical and 89.4% verbal aggression. Physical aggression more frequently occurred in nursing homes (83.9% of the employees) and verbal aggression was more common in the psychiatric clinic (96.7% of the employees). The proportion of the individuals affected in the workshop for people with disabilities was lower (41.9% and 77.4% respectively). The incidents impaired the physical (55%) and emotional well-being (77.2%) of the employees. The frequency of incidents (weekly: OR 2.7; 95% CI 1.1-6.4) combined with the lack of social support (OR 2.8; 95% CI 1.2-6.6) increased the probability of higher stress due to aggression.

**Conclusions:**

This study corroborates previous reports of frequent physical and verbal aggression towards care workers in the various areas of health care. The present study highlights differences between various areas of health care in Germany and the aggravating effect of prevention neglect such as missing social support at the workplace. Therefore our data suggest the need for improved target group specific prevention of aggressive incidents towards care workers and the need for effective aftercare in Germany.

## Background

Violence and aggression in the workplace is a significant problem in Germany and in other countries and is attracting increasing attention in public health research [1]. Violence and aggression are defined as: "Incidents where staff are abused, threatened or assaulted in circumstances related to their work"[2]. The present study examines violence and aggression used by patients towards nursing and health care staff. In this context, violence can include any form of verbal, physical and sexual aggression and/or physical violence.

Evidence suggests that nursing and health care staff frequently experience violence and aggression [3-5] resulting in potential impairment of physical and mental well-being in the affected person [6]. Furthermore, studies suggest that the consequences for the patients and the entire organisation are severe. Violence and aggression can, for example, negatively affect quality of care and treatment, cause longer periods of absenteeism and deteriorate the work climate [7].

A high number of unreported cases must be assumed, as only a fraction of the actual cases is reported [8,9]. Causes for the low number of reports given included the poor transparency of the reporting procedures and lack of support from superiors and a certain acceptance of violence and aggressive behaviour as being an integral part of nursing work [10].

Systematic research of the causes and consequences of aggression and violence towards employees in the health system is only just starting in Germany. Studies are often limited to investigations in mental health institutions; other fields of the health care system, as for example care for the elderly or care for people with disabilities, are still being neglected. Although international studies, e.g. from Scandinavia and the USA, frequently [3,4] show high levels of violence and aggression, it is only possible to apply their results to Germany to a limited extent. The reasons are, amongst others, to be found in the differing organisational structures of health care and in the different qualification standards of care staff [11]. Therefore the level of violence and aggression in the health care sector in Germany needs to be assessed in order to stipulate the development of measures to prevent violence and aggression and to deal with situations when they do arise [11,12].

There are also gaps in research regarding the prevention of violence and aggression and aftercare of incidents against nurses and health care workers. Although there are training programs for de-escalation management and further education measures for employees, systematic evaluation of such approaches are sparse [13,14]. In addition, there is a lack of care for the victims following aggressive incidents. Social support in the workplace and professional care provided by the respective institution are particularly important in order to ensure that the people affected come to terms with the experience and to combat the long-term consequences [2]. Once again little is known about the programs offered in Germany and their effectiveness.

It is therefore the aim of this study to examine the frequency, type and severity of verbal and physically aggression towards employees in nursing homes, psychiatric clinics and sheltered workshops and to investigate the consequences of such behaviour. Moreover, the availability of measures for dealing with aggression and violence at the workplace are analysed.

## Methods

### Study population

In a cross sectional retrospective survey, nursing staff and health care workers from four institutions of the region of Westphalia were interviewed in writing about their experience of violence in the last 12 months, using a standardised survey instrument. All verbal and physical attacks by patients were defined as an experience of aggression and violence. The terms aggression and violence were used interchangeably. An Internet search was performed to recruit the institutions, as this was considered a quick way to identify potential institutions with the respective contact people. The aim was to recruit institutions from different sections of the German health care system: facilities providing health care, nursing homes for the elderly and institutions offering care for the disabled. It makes sense to distinguish between these fields as each of them is concerned with very different kinds of patients, i.e. the frequency, type and consequences of violence and aggression will differ. Twenty institutions were approached, four of which participated in the study: two nursing homes for the elderly, a psychiatric clinic and a workshop for people with disabilities. At the start of the study, the project and its aims were personally explained to the institutions in order to increase the employees' motivation. The survey covered employees who are in frequent direct contact with the patients. In the areas surveyed, there were a number of other professional groups besides the actual nursing staff. Below, we distinguish between people working in the nursing/medical professions (nursing staff, therapists), those in pedagogic professions (educators, teachers, social education workers) and others (people spending a gap year doing voluntary work in the social sector, interns, and trainees). In total, 123 employees participated in the study. This corresponds to a response rate of 38.8%, which is a satisfactory outcome for a written survey. In the nursing homes the response rate was 32.0% and 15.0%, in the psychiatric clinic 50.8% and in the workshop for people with disabilities 60.0%. No information about the non-responders is available, hence, we cannot fully exclude a certain selection bias due to non-responders.

### Survey data

The experience with violence and aggressive behaviour and the measures used for managing such situations were recorded with a self-administered questionnaire, which is a modified and complemented version of the Staff Observation Scale Revised (SOAS-R) by Nijman and colleagues of the year 1999 [15]. Features of this instrument are its ease of handling and comprehensibility, and the psychometric quality. The questionnaire contains 20 questions from three blocks of topics. The first block covers questions about the participant and their profession, i.e. gender and age, the health care setting in which they work, their qualifications and experience at work. In the second part, their experience with violence and aggressive behaviour is surveyed. First they are asked separately and retrospectively about physically and verbally aggressive behaviour: "Have you experienced physically aggressive behaviour in the last twelve months? If so, how often?" and "Have you experienced verbal aggression in the last twelve months? If so, how often?" Further questions refer to the type and purpose of the aggressive behaviour, measures taken to contain aggressive behaviour and the recording of these cases. The last block of questions covers the need for ways to handle violence and aggression. It includes questions about physical and emotional consequences, general stress due to the incidents, help offers available in the institutions and their utilisation and social support.

### Ethical considerations

The participation in the study was voluntary and all participants gave their approval for the study and the data protection explanations by completing the questionnaires and returning them. It was not necessary to obtain the agreement of an ethics committee for the study as this is a survey which was analyzed in an anonymous manner. The participating institutions were told, prior to the study, that the study results would be published in a specialist journal in an anonymous form, to which they gave their consent.

### Data analysis

The data were recorded and analysed in SPSS version 13 [16]. Descriptive statistics were used to describe the sample. The 10-step scales for the assessment of stress, social support and preparation by the institution were recorded in 3-step scales: Poor or low (1-3), intermediate (4-7), good or high (8-10). Logistic regression was used to calculate odds ratios (OR) and 95% confidence intervals (CI) for putative factors which may influence stress perception. A dichotomous variable for stress perception was formed: 0 = not affected or grade of stress is low to intermediate (1-7); 1 = grade of stress is high (8-10 on the 10-step scale). The test level was specified as p < 0.05 and all tests are two-tailed.

## Results

The study population is described in table 1. Most of the participants work in nursing professions (74.8%). 51.2% of the participants have more than 15 years of work experience.

**Table 1:** Characteristics of the sample (n = 123)

		N	%
**Gender**	Male	44	35.8
	Female	79	64.2

**Age**	To 29 years	21	17.1
	30-39 years	35	28.5
	40-49 years	50	40.7
	Over 50 years	17	13.8

**Setting**	Nursing home	31	25.2
	Psychiatric clinic	61	49.6
	Workshop for people with disabilities	31	25.2

**Profession**	Nurses	92	74.8
	Social workers	22	17.9
	Others	9	7.3

**Work experience**	0 to 5 years	18	14.6
	6 to 10 years	18	14.6
	11 to 15 years	24	19.5
	Longer than 15 years	63	51.2

### Frequency of violence and aggression

**Figure 1:**
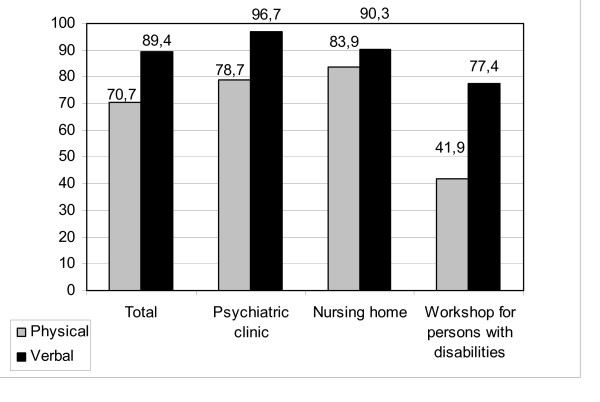
**Frequency of aggressive behaviour**. Fraction of employees who suffered physically or verbally aggressive behaviour in the last 12 months (classified by health care settings, n = 123).

In the twelve months prior to the survey, verbal aggression was experienced by 89.4% of the participants and physical aggression by 70.7% (figure 1). Employees in the workshop for people with disabilities (41.9%) were less affected by physical aggression than employees in other health care settings (78.7% in psychiatrist clinic and 83.9% in nursing homes). Nursing staff in particular are frequently exposed to physical aggression. 78.3% stated that they had been victims of physical aggression over the last twelve months, compared with 45.5% of those employed in pedagogic positions and 55.6% of those working in other fields (e.g. interns, people who spend a gap year doing voluntary work in the social sector, trainees). With regard to verbal aggression, there are no significant differences between the various professional groups.

Two-thirds of the participants (66.7%) experienced both forms of aggression, 23.6% experienced only verbal aggression and 4.1% only physical violence. Just 5.7% of the participants were not affected by incidents (no table). 44.8% of the participants experienced physical and 82.9% verbal aggression at least once monthly. 64 (52.0%) of the respondents suffered verbal or physical aggression at least once weekly.

### Reporting of the incidents

60.3% of the respondents reported the incidents with aggressive behaviour by patients. Most often the superiors were informed (97.1%). One-fifth of the afflicted respondents experienced incidents (20.0%) which were reported to the accident insurance (no table).

### Type of violence and aggression

The health care workers experienced abuse (92.2%), threatening gestures (59.2%), threats (55.2%), blows (47.4%), kicks (34.5%) or bites (26.7%). The use of objects (22.4%), sexual harassment (20.7%) and racial statements (11.2%) occurred less frequently. Further forms of aggression were scratching, pinching, spitting, boxing, shoving and self-harm. The target of the aggression was mostly the participant herself/himself (78.4%), followed by colleagues (59.5%), other patients, residents or employees (57.4%). In one quarter of cases, aggression towards objects or self-harm were recorded (no table).

### Consequences of violence and aggression

44.7% experienced physical impairment as a consequence of aggression (table 2). This most commonly led to short-term pain (60.0%). Six people (10.9%) received medical treatment after an assault, which corresponds to 4.9% of the whole study population. Anger or rage was reported by 58.5% of those who indicated emotional reactions. 42.3% of the respondents (54.7% of the affected people) reacted with insecurity, self-doubt and/or anxiety. After the experience of an assault, 22.0% of the respondents were tenser and more frightened at work (36.0% of the affected people). In individual cases, positive reactions, and an increase in empathy and sympathy were reported.

**Table 2:** Consequences of verbal and physical aggression for the study participants (n = 123)

	N	%
**Physical impairments**	**55**	44.7
**The following impairments were experienced**:		
Pain for less than about 10 minutes	33	60.0
Visible injury	27	49.1
Non-medical treatment required	27	49.1
Pain for more than about 10 minutes	16	29.1
Invisible injury	14	25.5
Medical treatment required	6	10.9

**Emotions**	**95**	77.2
**The following were experienced**:		
Anger, disappointment, rage	72	75.8
Helplessness	33	34.7
Anxiety, self-doubt, insecurity	52	54.7
Sadness	16	21.3
Other^a^	8	8.4

**Influence on the work**	**75**	61.0
**The following were experienced**:		
I am more careful	55	73.3
I am tenser, more anxious and have less fun at work	27	36.0
Other^b^	11	14.7

^a ^Exhaustion, tension, shock, sympathy, understanding, amazement

^b ^I am more empathetic, only uncertain for a short time and tense, I act more cautiously, I have more sympathy, I observe more clearly, I am more relaxed and attend to wait and see more, I am more absent-minded, I reflect more

116 people experienced verbal or physical aggression in the last twelve months before the survey. Of these, 36.2% felt slightly stressed, 36.2% felt moderately stressed and 27.6% felt highly stressed. 62.1% of the respondents felt that the social support offered at work to cope with such situations was good. However, only 30.1% of the respondents felt that the preparation for such situations provided by the institution was good (table 3). Colleagues are the most important source of social support (83.6%). Superiors (38.8%) and people outside work (25.0%) are less important. More men (32.6%) than women (13.9%) felt that they were badly supported (p-value = 0.05).

**Table 3:** Indications of stress due to verbal and physical aggression (n = 116), social support at work (n = 116) and preparation through the institutions (n = 123), separated according to health care settings

	Workshop for people with disabilities		**Nursing home**s		Psychiatric clinic		Total	
	**N**	**%**	**N**	**%**	**N**	**%**	**N**	**%**

**Stress due to aggression**	26		31		59		116	
Low	11	42.3	12	38.7	19	32.2	42	36.2
Intermediate	10	38.5	8	25.8	24	40.7	42	36.2
High	5	19.2	11	35.5	16	27.1	32	27.6

**Support at work**	26		31		59		116	
Poor	6	23.1	9	29.0	10	16.9	25	21.6
Intermediate	4	15.4	5	16.1	10	16.9	19	16.4
Good	16	61.5	17	54.8	39	66.1	72	62.1

**Preparation by the institution**	31		31		61		123	
Poor	8	25.8	13	41.9	14	23.0	35	28.5
Intermediate	13	41.9	12	38.7	26	42.6	51	41.5
Good	10	32.3	6	19.4	21	34.4	37	30.1

### Interventions

Most often interventions to stop the aggression took the form of discussions with the patient (81.0%), requests to change behaviour (58.6%), withdrawal from the patient (56.0%), requests for personal support (49.1%) and calm removal of the patient (47.4%). But more rigorous interventions were performed, too: Medication of the patient (46.6%), physical restraint (37.9%), forcible detention of the patient (33.6%) and forcible removal of the aggressive person (31.0%). 18.1% of the respondents asked for help from the police.

The employees reported that in their institutions training measures (59.3%) and case discussions (45.5%) were offered (table 4). The employees from the two nursing homes reported more often (67.7%) than their colleagues that such measures were not available (psychiatric clinic 8.2%, workshop for people with disabilities 9.7%, p-value = 0.001, no table). In the main, training measures and case discussions were used after incidents (37.9% or 40.5%). A significant proportion of the respondents did not take up any of the measures offered after such situations (37.9%). This was especially the case for the employees in the nursing home (54.8%). In the psychiatric clinic, 34.4% of the employees did not take up any of the measures offered after incidents. In the workshop for people with disabilities the proportion was 19.4% (p-value = 0.01).

**Table 4:** Information about offers for handling violence and aggression (n = 123)

	Offers in the institution		Utilisation after incidents	
	**N**	**%**	**N**	**%**

Training	73	59.3	41	37.9
Case discussions	56	45.5	47	40.5
Instructions for action	20	16.3	18	15.5
Aftercare discussions	23	18.7	21	18.1
Other offers	13	10.6	7	6.0
No offers	29	23.6	44	37.9

The extent of the impairment from verbal and physical aggression was assessed by participants on a scale from 1 to 10. The results were then compiled into three categories: low, medium and high levels of impairment. There were no differences between men and women (table 5). Age had no statistically significant influence on the perceived level of stress. The association between the stress level and the duration of employment was statistically significant. However, the odds ratios for the single categories of the period of employment are not statistically significant. The employees of the workshop for people with disabilities most rarely reported high impairments (16.1%). Here the difference between the settings is not statistically significant, either. The frequency of incidents (weekly: OR 2.7; 95% CI 1.1-6.4), and the lack of social support (OR 2.8; 95% CI 1.2-6.6) increased the probability for psychological strain through aggressive behaviour.

**Table 5:** Risk factors influencing stress in the people affected by violence and aggression

	Impairment through incidents					
	Low		High			
	N	%	N	%	OR	95%CI
**Women**	57	72.2	22	27.8	1	--
**Men**	34	77.3	10	22.7	0.6	0.3-1.6

**Age**						
20-29 years	17	81.0	4	19.0	1	--
30-39 years	26	74.3	9	25.7	1.9	0.5-8.1
40-49 years	35	70.0	15	30.0	1.6	0.4-5.9
≥ 50 years	13	76.5	4	23.5	1.8	0.3-9.5

Duration of employment *						
0-5 years	16	88.9	2	11.1	1	--
6-10 years	14	77.8	4	22.2	1.7	0.3-11.4
11-15 years	18	75.0	6	25.0	2.4	0.4-14.2
>15 years	43	68.3	20	31.7	2.9	0.6-14.8

**Setting**						
Nursing home	45	73.8	16	26.2	1	--
Psychiatric clinic	20	64.5	11	35.5	1.1	0.4-3.0
Workshop for people with disabilities	26	83.9	5	16.1	0.6	0.2-2.1

**Support**						
Good	64	81.0	15	19.0	1	--
Intermediate to poor	27	61.4	17	38.6	2.8	1.2-6.6

**Violence**						
Less frequent	49	83.1	10	16.9	1	--
Weekly	42	65.6	22	34.4	2.7	1.1-6.4

* Test for trend: p > 0.05

## Discussion

In literature, the prevalence of verbal and physical aggression towards health care staff ranges from 0.4% to 91% [4,17-20]. In the present study, 70.7% of the people interviewed experienced physical and 89.4% verbal aggression. Due to the very large range and differences between the different health systems studied, it is difficult to draw a comparison with other studies; however, the figures presented here underline the importance of the issue.

Studies performed in nursing homes give prevalence values between 11.4% and 40.0% [21,22]. In the present study, the high prevalence of violence and aggression towards staff of the two nursing homes is surprising. Here 83.9% of the employees suffered physical and 90.3% verbal aggression.

Employees in the psychiatric institution have to face physical and verbal aggression very often (78.7% and 96.7% respectively). The SOAS-R tool has already been used in a large number of studies covering psychiatric institutions. In the studies, the frequency of aggressive acts fluctuated between 0.4 and 89% [3,23]. Therefore the prevalence is not unexpected but still quite high.

Employees in workshops for people with disabilities suffered fewer physical (77.4%), and verbal incidents (41.9%) than workers in other institutions. To our knowledge, no other study covered this setting yet. These findings for workshops are comparable to those for residential homes. The percentage of those who experienced violence during their work in residential homes varies between around one third and 80% [24,25].

Apart from the nursing staff, the study also interviewed people working in other professions who have direct contact with the patients, with regard to their experience of violence and aggression. As described in other studies [26], nursing staff are the most prominent high-risk group in this study. But people in pedagogic positions or groups of people in other professions (e.g. interns) also report a high prevalence of aggressive acts. These results underline, on the one hand, how important it is to deal with the issue of violence and aggression towards nursing staff and, on the other hand, they show that other professional groups, who also have frequent contact with the patients, should not be neglected.

The negative influence of violence and aggression on the well-being of the affected person has been demonstrated in different studies. The consequences are emotions such as anger or anxiety [27] extending to addictions to substances [7] and psychological disorders such as burnout [28,29]. As physical injuries are mostly slight, the requirement for medical care is assessed as low [11,22]. However, our data indicate that many of the participants felt emotions like rage, anger, disappointment, helplessness and anxiety after incidents. As a consequence the employees reacted more tensely and more carefully. Indeed the general impairment due to physically and verbally aggressive behaviour was mostly classified as intermediate. But with the increase in incidents and poor social support, the perceived stress increased. The results of this study confirm the previous investigations and emphasise the need for action for psychological and organisational prevention and aftercare measures [13,30].

In the present study some health care workers reported that they reflected more often about their own actions or that they had increased empathy for the aggressors after the incidents. These findings indicate constructive management of the experience. They may be seen as resources for successful prevention of violence and aggression and care for the recipients following these events. More research on this aspect is needed.

The effectiveness of preventive measures which influence the frequency and extent of violence and aggression have only been examined in a few studies [13,14]. Although the respondents indicated that the institutions offered training measures or case discussions for managing violence and aggression, a large proportion of the workers felt that they were not very well prepared for situations with aggressive patients and people in care. In particular individuals employed in nursing homes felt that the facilities they worked with tended to leave them ill-prepared to deal with the aggression and violence shown by the patients. Therefore research on preventive interventions should be intensified and interventions must be better implemented in the organisations.

Apart from the offers provided within the organisations, it is important to take a close look at the social support offered in the workplace. Nursing staff members who are affected by violence and aggression seem to receive little social support, which, in turn, may have a negative effect on the way they cope with the incidents [30]. The study results support this assumption. The participants mainly received social support from their colleagues but only very rarely from their superiors. A further proportion of the study participants received no social support at all after incidents involving violence and aggression. This result is of major importance as the study also revealed that a lack of social support can increase the stress levels caused by such events.

A limitation of our study is the low response rate in the nursing homes. This might have introduced a selection bias if affected nurses were more likely to participate in the study. Therefore an overestimation of the prevalence rates in nursing homes cannot be ruled out. A further methodological limitation of this study is the relatively small sample of 123 people, allowing only for the detection of strong risk factors and limiting to generalization of the results. Further studies with larger populations are therefore needed in order to validate our results.

## Conclusions

For the first time a study has been conducted in Germany that analysed violence and aggression in various health care settings. In line with the literature, this study shows that violence and aggression towards workers are a major problem in the health care settings studied. Those caring for the elderly and for patients with mental problems are particularly exposed to violence and aggression. Insufficient social support increased the stress perceived after those incidences. Therefore our data suggest the need for improved target group specific prevention of aggressive incidents towards care workers and the need for effective aftercare in Germany. When conceiving such measures, a greater effort should be made to examine the differences between the various care sectors as well as between the different groups of individuals and professions.

## Competing interests

The authors declare that they have no competing interests.

## Authors' contributions

SF has made substantial contributions to conception and design, acquisition of data, and to the analysis and interpretation of the data. She has been involved in drafting the manuscript and gave her final approval of the version to be published. AZ made substantial contributions to the conception and design of the study and was involved in revising the manuscript critically for important intellectual content. She supervised the study and gave her final approval of the version to be published. AS participated in the study design and has made substantial contributions to the analysis and interpretation of data. SK has made substantial contributions to conception and design. AN has made substantial contributions to conception and design, and to the analysis and interpretation of data. He has been involved in drafting the manuscript and gave his final approval of the version to be published. All authors read and approved the final manuscript.

## Pre-publication history

The pre-publication history for this paper can be accessed here:

http://www.biomedcentral.com/1472-6963/10/51/prepub
